# Editorial: From Ecology to Brain Development: Bridging Separate Evolutionary Paradigms

**DOI:** 10.3389/fnins.2018.00447

**Published:** 2018-06-29

**Authors:** Francisco Aboitiz, Miguel L. Concha, Christian González-Billault, Jorge Mpodozis

**Affiliations:** ^1^Centro Interdisciplinario de Neurociencias, Escuela de Medicina, Pontificia Universidad Católica de Chile, Santiago, Chile; ^2^Faculty of Medicine, Institute of Biomedical Sciences, Universidad de Chile, Santiago, Chile; ^3^Biomedical Neuroscience Institute, Santiago, Chile; ^4^Geroscience Center for Brain Health and Metabolism, Santiago, Chile; ^5^Laboratory of Cell and Neuronal Dynamics, Department of Biology, Faculty of Sciences, Universidad de Chile, Santiago, Chile; ^6^The Buck Institute for Research on Aging, Novato, CA, United States; ^7^Laboratorio de Neurobiología y Biología del Conocer, Departamento de Biología, Facultad de Ciencias, Universidad de Chile, Santiago, Chile

**Keywords:** critical periods, computational neuroscience, visual perception, microcircuits, homology, birds, cerebral cortex, olfaction

This special topic proposes an integrative view of brain evolution involving ecology, behavior, cognition and neurodevelopmental processes. We address three main questions, (i) the role of sensorimotor systems in brain evolution, (ii) the evolution of computational capacities and neural circuits, and (iii) the role of development in shaping brain evolution.

## Evolution of sensorimotor systems

The organism interacts with the environment through a sensory-motor interface, which is critical for driving evolutionary change. Pallas discusses the relation between embryonic processes that build up sensory processing structures in mammals, and the selection of specific developmental pathways yielding adaptive neuronal networks. Pallas focuses on critical periods of sensory development, where more rigid phenotypes may be selected in predictable environments while developmental flexibility is favored in conditions of unpredictability. Wylie et al. address the evolution of the visual system in birds in the context of sensory adaptations of different species. For example, components of the accessory optic system involved in the analysis of optic flow are particularly developed in hummingbirds, while the Wulst, a brain component supporting binocular vision, is enlarged in frontal looking species like owls. Aboitiz and Montiel focus on the role of olfaction in the origin of the mammalian neocortex. The latter is proposed to develop as an interface between olfactory and hippocampal networks involved in navigation, recruiting additional sensory inputs into this orientation network. Aboitiz then discusses the evolutionary origin of the human speech networks, from the peripheral control of the vocal system to the central networks controlling auditory-vocal coordination. A key innovation in human evolution is the development of an auditoy-vocal cortical network that increases vocal working memory and vocal learning capacity. Ending this section, Ravignani reviews the topic of behavioral antisynchrony in interindividual coordination, as seen in two disparate species: fiddler crabs and human infants. Ravignani proposes a broad framework to interpret these behaviors, relying on the evolution of perceptual biases driving animals toward rhythmic coordination.

## Cognitive computations and neural circuits

A second level is the generation of internal processing devices that modulate the relation between perception and behavior. King discusses the ecological underpinnings of cognitive computation, warning that computational analogies of the human mind are rooted in the early conceptual work of George Boole, long before the technological digital revolution came to be. In a different approach, Bosman and Aboitiz take issue with the extended conservation of brain microcircuits, from crayfish to mammals. Canonical microcircuits can be described in several taxa and neural systems, consisting of input-receiving neurons, output neurons and excitatory and inhibitory interneurons that regulate the balance between excitation and inhibition in larger neural networks. Finally, Faunes et al. discuss homology of brain components across species, arguing that hodology (neural connectivity) is the most relevant criterion to establish homologies, as opposed to genetic criteria. As a critical example, they propose homology between a globular brain structure termed dorsal ventricular ridge (DVR) in reptiles/birds, and parts of the six-layered mammalian neocortex, on the basis that similar sensory projections ascending from brainstem nuclei synapse in both structures.

## Development and brain evolution

Evolution is a sequence of ontogenies rather than of adult states, and change must take place through developmental transformations. In contrast to the hodological perspective mentioned above, some studies propose non-homology between the neocortex and the DVR, as they derive from different embryonic components, the dorsal and the ventral pallium respectively. Instead of focusing on embryonic compartments, Montiel and Aboitiz look for underlying developmental mechanisms that modulate brain patterning in reptiles/birds and mammals, proposing differential regulation of specific morphogenetic signals in these two groups. The laminar mammalian neocortex would have developed from upregulation of morphogens originating in the dorsal hemisphere, while in reptiles/birds these factors remained downregulated yielding a globular DVR. Other patterning signals like the gene Pax6 are modulated in both groups. Luzzati also touches on the problem of the origin of the mammalian neocortex, evidencing a similarity between cellular phenotypes in superficial neocortical layers with those found in reptilian brains. Luzzati argues for a superposition between the dorsal cortex and the olfactory cortex in the evolutionary emergence of the neocortex. In the last article in this section, Salas et al.discuss ontogenetic brain scaling in lampreys, one of the two living jawless vertebrates. Salas and collaborators assess brain and body growth in the lamprey's ontogeny, in order to test the hypothesis that the developmental transitions in behavior are related to distinct events in the development of specific brain components. Particularly, brain size increases markedly in the metamorphosis, as opposed to body size that remains unchanged in this process.

Our aim in this Topic has been to show research that bridges two approaches that have been difficult to reconcile, one that focuses on the evolution of behavior and brain function, and the other that is concerned with the developmental mechanisms involved in the production of new phenotypes. The presumed homology between the mammalian neocortex and the reptilian/avian DVR is an eloquent example of this, being perhaps the most controversial problem of modern comparative neuroanatomy. In one perspective, (i) there was transformation of an ancestral DVR-like structure into a cortical, layered morphology during mammalian evolution, concomitant with (ii) a topographic reorganization of the embryonic brain to place the DVR adjacent to the dorsal pallium (Faunes et al.). In the other perspective, (i) the neocortex and the DVR are not comparable because they belong to different embryonic components, and (ii) mesencephalic sensory axons were redirected in mammals from a ventral position into the dorsal pallium where the neocortex develops. While Montiel and Aboitiz favor the second proposal, both scenarios may be compatible with an increase in dorsalization signals that impose a laminar organization to the mammalian neocortex.

We hope that this initiative will contribute to view the nervous system as a unified system, subject to both functional and developmental constraints, where evolution results from the interplay of these different factors.

## Author contributions

All authors listed have made a substantial, direct and intellectual contribution to the work, and approved it for publication.

### Conflict of interest statement

The authors declare that the research was conducted in the absence of any commercial or financial relationships that could be construed as a potential conflict of interest.

